# Molecular docking and experimental validation of the effect of ergothioneine on heat shock protein-70 following endurance exercise by Arabian stallions

**DOI:** 10.1186/s12917-023-03584-6

**Published:** 2023-01-30

**Authors:** Adakole Sylvanus Adah, Joseph Olusegun Ayo, Deborah Arimie Adah, Charles Obiora Nwonuma, Teslim Alabi Lawal

**Affiliations:** 1 Department of Veterinary Physiology and Biochemistry, University of Ilorin, Ilorin, Nigeria; 2 Department of Veterinary Physiology, Ahmadu Bello University, Zaria, Nigeria; 3 Department of Veterinary Medicine, University of Ilorin, Ilorin, Nigeria; 4 Department of Biochemistry, Landmark University, Omuaran, Nigeria; 5 Computational Biophysical Laboratory, Department of Pure and Applied Chemistry, Ladoke Akintola University, Ogbomoso, Nigeria

**Keywords:** Arabian stallion, Ergothioneine, Heat shock protein-70, Molecular docking, Oxidative stress

## Abstract

**Background:**

Exercise-induced oxidative stress is a challenge in equine sports. This study aims at determining the effects of ergothioneine on heat shock protein-70 (HSP-70) following the stress of an endurance exercise of 30 km by Arabian stallions. Molecular docking was also done to investigate the interaction between the ligand ergothioneine and heat shock protein-70 using sulfogalactosylceramide and sulfogalactoglycerolipid as standards.

The study involved a total of 18 clinically healthy stallions, with an average age of 6.7 ± 2.4 years and an average weight of 411.54 ± 12.46 kg. Only clinically healthy stallions were selected as subjects. The stallions were divided into two groups of nine stallions each. Group I (ERGX) was administered ergothioneine at a dose of 0.02 mg/kg once daily orally for four weeks while group II (ERGN) was not administered ergothioneine. The activities of the antioxidant enzymes superoxide dismutase, catalase, and glutathione peroxidase were determined in the two groups before and post-exercise. The concentrations of malondialdehyde and HSP-70 were also determined.

**Results:**

The results obtained showed that the activities of the antioxidant enzymes and concentration of HSP-70 were higher (*P* < 0.05) in the ERGX group compared to the ERGN group. The concentration of malondialdehyde was however lower in the ERGX group. Following molecular docking, ergothioneine and the selected standards have common amino acids at the site of interaction with the target protein (HSP-70) suggesting that ergothioneine may have a modulatory effect on the synthesis of HSP-70.

**Conclusion:**

The results obtained indicated that ergothioneine modulated the synthesis of HSP-70 and the biomarkers of oxidative stress. It was therefore concluded that ergothioneine may be beneficial to horses subjected to endurance exercise.

## Background

It is widely known that an increase in the generation of free radicals and reactive oxygen species (ROS) during exercise has both beneficial and detrimental physiological consequences [1]. Exercise increases oxygen demand, especially in skeletal muscle, which dramatically alters the blood flow to different organs. Additionally, exercise-induced muscle damage promotes neutrophils and macrophages to infiltrate the area of injury causing physiological changes, which boost the formation of free radicals and cause oxidative damage to biomolecules. However, tissue growth is regulated by the free radicals produced during exercise. Mild oxidative stress may also help the body combat inflammation and infection [2, 3].

All animals including horses naturally produce reactive oxygen species, reactive nitrogen species, and sometimes reactive chlorine species as part of their physiological processes through normal cellular operation [4]. Oxidative damage, also known as oxidative stress, is a term frequently used to imply random, indiscriminate damage to a wide range of biomolecules when an excess of these free radicals cannot be processed gradually or in cases of poor availability of the naturally occurring antioxidant for protection of the body [5]. Exercise-induced oxidative stress affects mitochondrial biogenesis, mitochondrial dynamics (i.e., fission and fusion), and mitochondrial turnover (i.e., mitophagy) in skeletal muscle as reported by Joseph et al. [6].

The primary function of nutritional antioxidants such as ergothioneine is to scavenge free radicals. They do this in four ways: 1) by neutralizing free radicals, 2) by repairing oxidized membranes, 3) by reducing the production of reactive oxygen species, and 4) by neutralizing reactive oxygen species through lipid metabolism [7]. Exogenous antioxidants have received significant attention for their potential to prevent or reduce oxidative stress, minimize physical stress and muscle pain, and improve athletic performance [8, 9].

Stress proteins, also known as heat-shock proteins (HSPs), are found in all the cells of all living organisms. Specific HSPs such as HSP 70, are essential for the folding and unfolding of proteins [10–12]. The molecular chaperone's central hub, heat-Shock Protein-70 (HSP-70), regulates protein homeostasis in the ATP-containing compartments of animal cells. [13]. It is the most abundant and temperature-sensitive of all the HSPs and plays an important role in environmental, exercise, and heat stress responses [14].

Molecular docking is a process through which small molecules are docked into the macromolecular structures for scoring their complementary values at the binding sites. It is a vibrant research area with dynamic utility in structure-based drug designing, lead optimization, biochemical pathway, and for drug designing being the most attractive tools. Two pillars for a successful docking experiment are correct pose and affinity prediction [15].

Ergothioneine is a unique, naturally occurring antioxidant synthesized from the amino acid histidine that can only be found in food, and cannot be synthesized by higher animals. The avidity with which it is absorbed by tissues, the specific and profound influence it has on cellular processes, and the degree to which cells store it suggest that this molecule may have a significant physiological role [16]. Ergothioneine directly scavenges free radicals and reactive oxygen species (ROS), such as hypochlorite acid and peroxynitrite, in addition to down-regulating the generation of free radicals such as hydroxyl ions [17]. Furthermore, it enhances other natural antioxidant defense mechanisms, activating a MAPK-based internal antioxidant pathway and regulating the activities of peroxidase and antioxidant enzymes such as superoxide dismutase [16]. In contrast to other thiol compounds, ergothioneine chelates a variety of divalent metal cations, such as Fe, Cu, Zn, Ni, and Co, creating an ergothioneine-metal complex that lowers the metal ions' oxidative reactivity [17].

This study aims to validate by experiment and molecular docking the effect of ergothioneine on heat shock protein-70 following endurance exercise in Arabian stallions.

## Methods

### Experimental site and animals

The experiment was carried out at Ilorin, Nigeria (8.4799° N, 4.5418° E), during the hot-dry season (March 2022). Ilorin is a city in the Guinea Savannah region. The study involved a total of 18 healthy stallions (*Equus ferus caballus*) of the Arabian breed, with an average age of 6.7 ± 2.4 years and an average weight of 411.54 ± 12.46 kg. Only clinically healthy stallions were selected as subjects. The stallions underwent clinical screening for parasites such as trypanosomosis, babesiosis, strongylosis, and common horse infections such as equine infectious anaemia and equine influenza (details in Table 1). The stallions were kept in facilities with cement block walls and corrugated iron roofing. They received hay together with high-energy concentrate as food. Water was accessible at all times by the stallions. The stallions' weights were measured using a standardized weight measuring tape (Zoometric Tape, Hauptner, Dietlikon-Zurich, Switzerland), and weights were estimated using the formula:

**Table 1:** Diseases of horses that were screened

Diseases	Aetiology	Screening Method
Trypanosomosis	*Trypanosoma* Spp	Checked blood smear for the presence of *Trypanosoma* Spp
Babesiosis	*Babesia* Spp	Checked blood smear for parasite for *Babesia* Spp
Helminth parasitic diseases e.g. Strongylosis	Nematodes e.g.*Strongylus vulgaris*	Faecal examination for the presence of eggs of helminth using floatation technique
Equine infectious anaemia	Equine infectious anaemia virus	Determination of packed cell volume of blood to check for anaemia and also checked for antibodies against the disease in the blood using the Coggins test
Equine influenza Bacterial Infections e.g Strangles	Equine influenza virus Bacteria species e.g. *Streptococcus equi*	Clinical signs of respiratory origin, vital parameters Taking vital parameters, physical examination and bacteria culture of faecal samples, discharges, and swabs

Body weight (in kilograms) = [girth circumference squared (in centimeters) x body length (in centimeters)] / 11,877.

Their ages were obtained from records kept at the stable. The stallions came from a royal stable in Ilorin, Nigeria (8.4799°N, 4.5418°E), which is in the Guinea Savannah region and has high humidity and rainfall for nearly nine months of the year. They were chosen by simple random sampling. The stallions were divided into two groups of nine stallions each by randomization. Group I (ERGX) was administered with ergothioneine while group II (ERGN) was not administered with ergothioneine.

### Exercise protocol

All stallions were trained for endurance exercise before the trial began by subjecting them to a calibrated exercise routine every day for four weeks. On the day of the experiment, each stallion was mounted by an experienced rider who is approximately 10% of the stallion’s body weight and subjected to an endurance exercise of 30 km. At the end of every 10 km during the exercise, the stallions rested for 15 min and were provided with cool water.

### Administration of ergothioneine to stallions

Each stallion in group I was administered ergothioneine at the dose of 0.02 mg/kg daily by oral administration for four weeks. Briefly, ergothioneine was dissolved in sterile, deionized water and aspirated into an appropriate syringe, and administered to the stallions before feeding in the morning.

### Blood sampling

Each horse had 10 mL of blood drawn from the jugular vein before and after the exercise. The blood was drawn into plain sample tubes, to harvest serum. The collected blood samples were transported to the lab in a Coleman box containing ice, where they were analysed immediately.

Assay for Biochemical Parameters (Summarised in Table 2).

**Table 2:** Method used to determine biochemical parameters

Parameters	Methods of determination
Superoxide dismutase	Spectrophotometric method as described by Misra and Fridovich, [18]
Catalase	Spectrophotometric method as described by Aebi [20]
Glutathione peroxidase	Spectrophotometric method as described by Sedaghatfard et al. [19]
Malondialdehyde	Spectrophotometric method as described by Draper and Hadley, [21]

### Determination of the activity of superoxide dismutase

The activity of the enzyme superoxide dismutase (SOD) was determined as described by Misra and Fridovich, [18]. Briefly, the spectrophotometric technique was used to measure SOD activity. The experimental and reference reaction sets were conducted simultaneously. The reference tubes held all the reagents except the enzyme source, while the experimental tubes contained 0.2 ml Nitro blue tetrazolium (NBT), 0.2 ml phenozine methosulphate (PMS), 1.1 ml sodium pyrophosphate buffer, and 20 µl enzyme sources. After adding 0.2 ml of reduced-nicotine adenine dinucleotide, both reactions began at the same time (NADH). Each tube contained 1.0 ml of glacial acetic acid to terminate the reaction after 90 s, while the reference tubes contained 20 µl of the enzyme source. The absorbance was measured at 560 nm. The SOD enzyme activity, measured in units/L of serum, was defined as the quantity of enzyme generating half the maximal inhibition of NBT reduction.

### Determination of the activity of glutathione peroxidase

The activity of the enzyme glutathione peroxidase was evaluated with glutathione peroxidase detection kit (Ransel kit produced by Randox Co.). Glutathione peroxidase catalyses the oxidation of glutathione (GSH). In the presence of glutathione reductase (GR) and NADPH, the oxidized glutathione (GSSG) was immediately converted to the reduced form, with concomitant oxidation of NADPH to NADP^+.^ The absorbance at 340 nm against blank was measured spectrophotometrically. One unit (U) of glutathione peroxidase activity was defined as the amount of enzyme that converts 1 μmol of NADPH to NADP^+^ per minute. The glutathione peroxidase activity was expressed as unit per litre of serum (U/L) as described by Sedaghatfard et al. [19],

### Determination of the activity of catalase

Serum catalase activity was assayed spectrophotometrically by monitoring the decomposition of hydrogen peroxide (H_2_O_2_) using the procedure described by Aebi [20]. Briefly, 0.5 mL of 30 mmol/L H_2_O_2_ solution in 50 mmol/l phosphate buffer (pH = 7.0) was prepared and 1 ml of 1:10 diluted serum supernatant was added and the consumption of H_2_O_2_ was measured spectrophotometrically at 240 nm for 2 min at 25ºC. Catalase activity was expressed as the unit consumed/min per litre of serum.

### Measurement of lipid peroxidation (Malondialdehyde concentration)

Malondialdehyde concentration was evaluated using the method of Draper and Hadley, [21]. To evaluate lipid peroxidation, a modified high-performance liquid chromatography method was used which is based on the reaction of malondialdehyde (MDA) with thiobarbituric acid (TBA) to form a coloured MDA-TBA adduct. Briefly, 0.5 mL serum supernatant was added to a 2 mL TBA reagent containing 0.375% TBA, 15% trichloroacetic acid, and 0.25 mol/L HCl. The mixture was immediately heated (60 min at 95 ^0^C) and cooled with running water. Thereafter, butanol-pyridine (15:1, v/v) (1 mL) was added and the final volume was adjusted to 2 mL with distilled water. After vigorous mixing, the organic layer was separated by centrifugation [16000 g, 3 min, at room temperature (25–26 °C)]. The supernatant was analysed on a UV–visible spectrophotometer fitted with an 80 μL flow cell. The absorbance was measured at 532 nm. 1, 1, 3, 3-tetra ethoxy propane was used as a standard, and values of MDA-TBA reactive substances were expressed as µmol per litre of serum.

### Determination of heat shock protein-70

The serum HSP-70 concentration was determined using the horse HSP-70 ELISA detection kit produced by My BioSource (San Diego, California, United States). It is an ELISA (enzyme-linked immunosorbent assay) kit for measuring the presence of Heat-shock Protein 70 in microwell, strip plate format (HSP-70). A colorimetric detection method was used to identify HSP-70 antigen targets in serum using the ELISA analytical biochemical technique of the kit, which was based on HSP-70 antibody-HSP-70 antigen interaction. The inter-assay variation of the Elisa kit was 6.2% while the intra-assay variation was 5.8%.

### Molecular docking protocol

#### Retrieval and preparation of the ligand and standard drugs

The ligand (ergothioneine) and the selected standard compounds sulfogalactosylceramide and sulfogalactoglycerolipid (selected as standards because they are found naturally in the body and interact with HSP-70). They were retrieved from the compound data bank in their three-dimensional format. These phytochemicals were converted to a pre-docking tool readable format (.pdb) using the Discovery Studio tool.

### Retrieval and preparation of the target receptor

A 3D structure of a Heat Shock Protein (HSP-70; PDB: 1X3S) was retrieved from the Protein Data Bank (www.rcsb.org). To prevent unwanted interference with the molecular docking simulation, residues such as water, ligands, and heteroatoms that are attached to the protein were removed to have its crystal form by using Discovery studio 2019 [22].

### Molecular docking

The molecular docking, a structure-based drug design tool was employed to forecast the interactions that occur between the target protein (HSP-70; PDB: 1X3S) at the binding site, the selected ligand (ergothioneine), and the standard drugs (sulfogalactosylceramide and sulfogalactoglycerolipid) using AutoDock Vina (MGL tools-1.5.6) and Biovia Discovery studio 4.5. The docking compatible (pdbqt) files of the ligand, standard drugs, and the target protein were created using the AutoDock tool with the grid sizes of 68, 64, and 64 for the x, y, and z axes; grid centers of 17.349 × 28.677 × 16.059, respectively, and 1.000 Å spacing. The binding affinity of each phytochemical was used to predict their inhibition constants (K_i_) using Eqs. 1 and 2 [22, 23].

1 $$\Delta G\;=\;-RT\ln K_i$$

2 $$i=e\left[\frac{-\triangle G}{RT}\right]$$

where R = Gas constant (1.987 × 10^–3^ kcal/mol); T = 298.15 K (absolute temperature); Ki = Inhibition constant; ΔG = Binding energy/affinity.

### Data analysis

The data obtained from this study were expressed as mean ± SEM and checked whether it is normally distributed using the Shapiro–Wilk test. The data were discovered to be normally distributed. The data were analysed using the student’s *t*-test. Values of P < 0.05 were considered to be significant. All data were analysed using the software GraphPad Prism (version 5.3) (San Diego, California USA).

## Results

The activities of the antioxidant enzymes; superoxide dismutase, catalase, and glutathione peroxidase were significantly higher (*P* < 0.05) in the ERGX group compared to the ERGN group (Table 2). The concentration of HSP-70 was also higher (*P* < 0.05) in the ERGX group compared to the ERGN group. Malondialdehyde concentration was, however, significantly lower (*P* < 0.05) in the ERGX group (Table 3).

**Table 3:** Biochemical parameters of stallions

Parameters		ERGN	ERGX
Superoxide dismutase (u/ml)	Pre-exercise	1.62 ± 0.21	2.23 ± 0.31^1^
	Post-exercise	2.49 ± 0.14^a^	7.54 ± 4.02^b2^
Catalase (u/ml)	Pre-exercise	4.97 ± 0.23	4.87 ± 0.57^1^
	Post-exercise	6.04 ± 0.43^a^	9.76 ± 4.91^b2^
Glutathione peroxidase (U/L)	Pre-exercise	259.90 ± 6.32	287.23 ± 9.78^1^
	Post-exercise	278.13 ± 8.43^a^	318.08 ± 18.86^b2^
Glutathione reductase (U/L)	Pre-exercise	0.44 ± 0.16	0.45 ± 0.03
	Post-exercise	0.87 ± 0.34	1.58 ± 1.33
Malondialdehyde (µmol/L)	Pre-exercise	5.61 ± 0.34	5.32 ± 1.06^1^
	Post-exercise	10.24 ± 5.77^b2^	7.26 ± 0.56^a^
Heat shock protein-70 (ng/ml)	Pre-exercise Post-exercise	237.16 ± 10.91 284.38 ± 18.71^a^	242.31 ± 12.03 307.86 ± 24.98^b^

^a,b^Means for the same row having different superscript letters are significantly (*P* < 0.05) different. ^1,2^Means for the same column having different superscript numbers are significantly (*P* < 0.05) different

*ERGN* Group not administered with ergothioneine, *ERGX* Group administered with ergothioneine

The docking result presented in Table 4 shows that ergothioneine has a relatively close binding affinity (-4.8 kcal/mol) to the selected standard (sulfogalactosylceramide: -6.0 kcal/mol and sulfogalactoglycerolipid: -6.4 kcal/mol).

**Table 4:** Binding properties of ergothioneine and selected standards (sulfogalactosylceramide and sulfogalactoglycerolipid

Ligands	Binding affinity	6LU7 Receptor amino acids forming H-bond with ligands	Electrostatic/ Hydrophobic interactions involved	Inhibition Constant
Ergothioneine	-4.8	His227, Asp232	Arg72, Asp69, Glu231	304.1
Sulfogalactosylceramide	-6.0	Arg264, Asn235, Ser254	Trp90, His89, Glu231, Lys257	40.2
Sulfogalactoglycerolipid	-6.8	Asp292, His227, Glu231, Arg72	Asn235	20.4

*Trp* Tryptophan, *His* Histidine, *Asp* Aspartate, *Glu* Glutamate, *Arg* Arginine, *Ser* Serine, *Ans* Asparagine, *Lys* Lysine

**Fig. 1:**
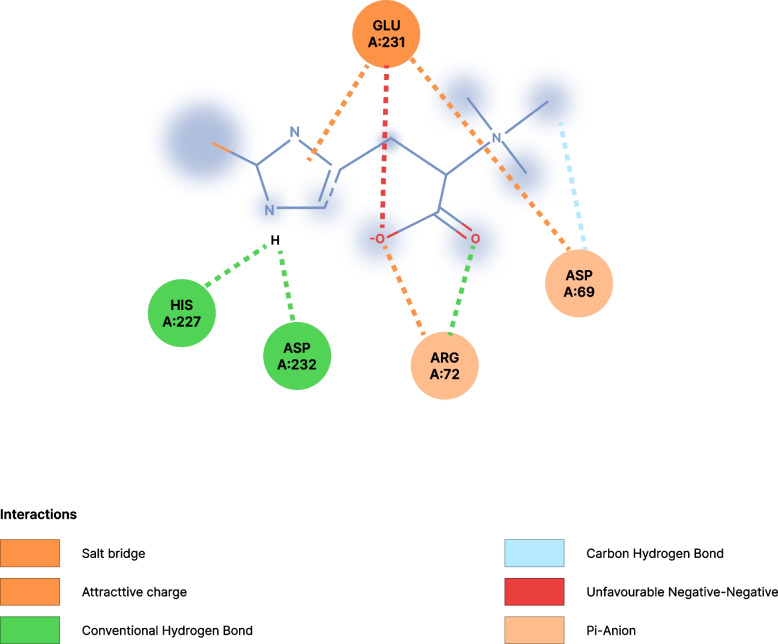
Interaction between ergothioneine and HSP-70. *His* Histidine, *Asp* Aspartate, *Glu* Glutamate, *Arg* Arginine, *Ans* Asparagine

**Fig. 2:**
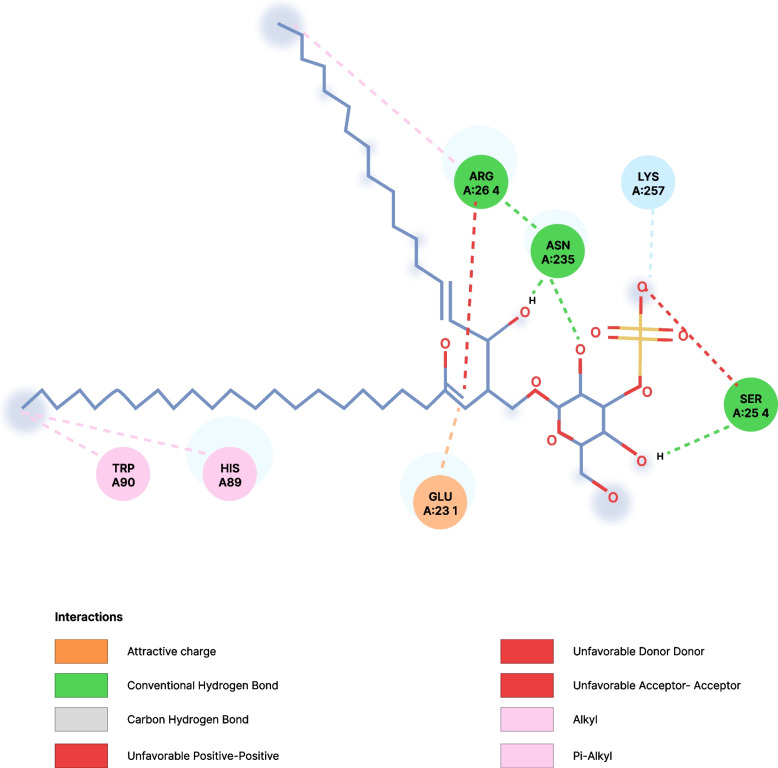
Interaction between Sulfogalactosylceramide (standard) and HSP-70. *Trp* Tryptophan, *His* Histidine, *Asp* Aspartate, *Glu* Glutamate, *Arg* Arginine, *Ser* Serine, *Ans* Asparagine, *Lys* Lysine

**Fig. 3:**
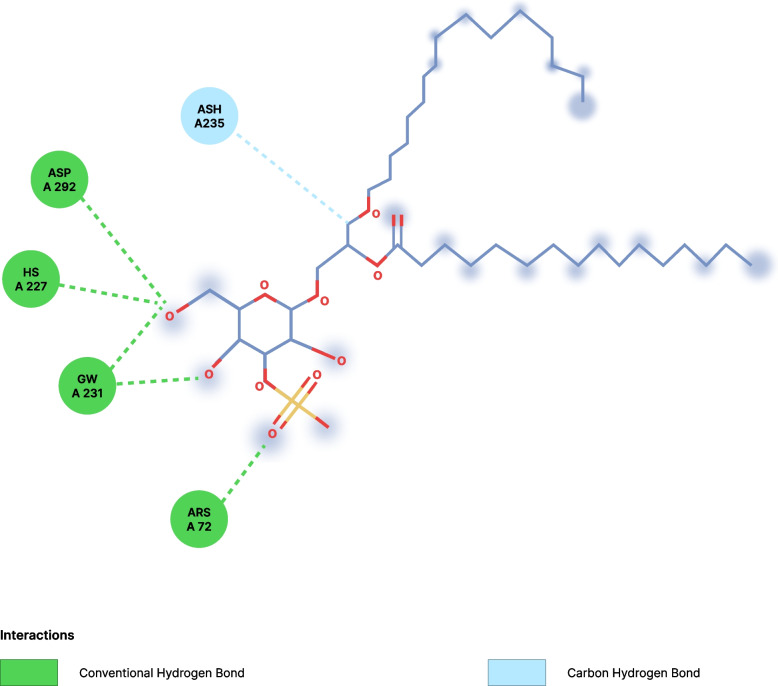
Interaction between Sulfogalactoglycerolipid and HSP-70. *His* Histidine, *Asp* Aspartate, *Glu* Glutamate, *Arg* Arginine, *Ans* Asparagine

The interactions of ergothioneine, sulfogalactosylceramide, and sulfogalactoglycerolipid with the target protein (HSP-70) are shown in Figs. 1, 2, and 3 respectively.

## Discussion

Heat-shock protein 70 being the most abundant and temperature-sensitive heat-shock protein plays an important role in environmental, exercise, and heat stress responses [14, 24]. In addition to their stress-related functions, HSP-70 performs many other functions including folding and maturation of proteins, and translocation of proteins across tiny membrane pores into organelles, such as the endoplasmic reticulum, and mitochondria [25, 26]. In this study, we observed a significantly higher concentration of HSP-70 in the experimental group (ERGX) compared to the control group (ERGN) suggesting some modulatory effects of ergothioneine. This finding is similar to the report of Sakrak et al. [27] who reported a higher concentration of HSP-70 following the administration of ergothioneine in experimental rats. Additionally, ergothioneine supplementation before exercise appears to activate the HSP-70 synthesis pathways as seen in this study. An upregulation of HSP-70 and a higher expression of HSP-70 as observed in the ERGX group suggests protection against protein misfolding disorders, systemic inflammation, and muscular dysfunction and also acts as a preventive measure against motor neuron and muscle cell death because HSP-70 response to exercise may serve as one of the stabilizers of disordered myofibrillar structures and contribute to the repairs and adaption processes of muscles following the stress of exercise [28–30]. However, the exact mechanism by which ergothioneine modulates HSP-70 is not yet understood and requires further investigation.

Interestingly post-exercise, the activities of superoxide dismutase, catalase, and glutathione peroxidase were observed to be significantly higher in the ERGX group compared to the ERGN group suggesting a modulatory role by ergothioneine. From this present study, it is evident that the pre-treatment of the experimental group with ergothioneine before exercise protected the body of the stallions by a downward regulation in the production of reactive oxygen species as evidenced by the higher activities of the antioxidant enzymes in the ERGX group [31, 32]. This is because, under conditions of oxidative stress or inflammation, ergothioneine has been reported to be potent as a cell-protective molecule and a potent antioxidant [16]. Ergothioneine has also been reported by Fu and Shen [33] to have a positive correlation with glutathione peroxidase during oxidative insults as these molecules play a crucial role in the body's antioxidant defense against oxidative stress. In addition, the superoxide dismutase enzyme has been reported to reduce inflammation and prevent the onset of precancerous cell changes due to stressful conditions [34–36]. In this study, higher activities of these enzymes were recorded in group I (ERGX group) suggesting a modulatory role by ergothioneine.

As a stable by-product of lipid peroxidation, malondialdehyde is commonly utilized as a biomarker of lipid peroxidation [37]. Lipid peroxidation increases significantly during oxidative stress. In this study, we observed a lower concentration of MDA in the ERGX group further confirming the antioxidant role of ergothioneine in the group.

The identification and investigation of protein–ligand interactions are crucial in the testing of medications and dietary supplements to increase the ligand affinity to the pocket through optimization of the selected drug to improve its properties [22, 38].

The molecular docking shows that ergothioneine interacted with the receptor through conventional hydrogen bonds with His227 and Asp232, and through Pi-Anion with Arg72, Asp69, and Glu231 as shown in Table 4. Sulfogalactosylceramide formed bonds with the target protein via conventional hydrogen bonds with Arg264, Asn235, and Ser254, Pi-Alkyl interactions with Trp 90 and His 89, carbon-hydrogen bond with LYS 257, and attractive charge interactions with GLU 231. Similarly, sulfogalactoglycerolipid interacted with the target protein at the active site via a conventional hydrogen bond with Asp292, His227, Glu231, and Arg72, as well as via a carbon-hydrogen bond with ASN 235. Interestingly, ergothioneine and the selected standards have one or more common amino acids at the site of interaction with the target protein as shown in the figures below. This further buttressed the enhancement claim of ergothioneine of the target protein (HSP-70). A limitation of this study is that the exact mechanism by which ergothioneine modulates HSP-70, biomarkers of oxidative stress is not well understood and requires further investigation.

It was therefore concluded that the administration of ergothioneine before exercise resulted in an upregulation of HSP-70 in the horses studied, therefore ergothioneine will be beneficial to horses subjected to the stress of exercise. However, the exact mechanism by which this upregulation occurred is not well understood and therefore requires further study.

## Data Availability

The datasets used in this study are available from the corresponding author upon request.

## Abbreviations

ERGX: Group administered with ergothioneine
ERGN: Group not administered with ergothioneine
HSP-70: Heat shock protein 70
Trp: Tryptophan
His: Histidine
Asp: Aspartate
Glu: Glutamate
Arg: Arginine
Ser: Serine
Ans: Asparagine
Lys: Lysine
ROS: Reactive oxygen species
